# Piezoelectric BiFeO_3_ Thin Films: Optimization of MOCVD Process on Si

**DOI:** 10.3390/nano10040630

**Published:** 2020-03-28

**Authors:** Quentin Micard, Guglielmo Guido Condorelli, Graziella Malandrino

**Affiliations:** Dipartimento di Scienze Chimiche, Università degli Studi di Catania, INSTM UdR Catania, Viale Andrea Doria 6, I-95125 Catania, Italy; qmicard@gmail.com (Q.M.); guido.condorelli@unict.it (G.G.C.)

**Keywords:** BiFeO_3_, MOCVD, Si substrate, thin film, perovskite, lead-free piezoelectric, energy harvesting

## Abstract

This paper presents a simple and optimized metal organic chemical vapor deposition (MOCVD) protocol for the deposition of perovskite BiFeO_3_ films on silicon-based substrates, in order to move toward the next generation of lead-free hybrid energy harvesters. A bi-metal mixture that is composed of Bi(phenyl)_3_, and Fe(tmhd)_3_ has been used as a precursor source. BiFeO_3_ films have been grown by MOCVD on IrO_2_/Si substrates, in which the conductive IrO_2_ functions as a bottom electrode and a buffer layer. BiFeO_3_ films have been analyzed by X-ray diffraction (XRD) for structural characterization and by field-emission scanning electron microscopy (FE-SEM) coupled with energy dispersive X-ray (EDX) analysis for the morphological and chemical characterizations, respectively. These studies have shown that the deposited films are polycrystalline, pure BiFeO_3_ phase highly homogenous in morphology and composition all over the entire substrate surface. Piezoelectric force microscopy (PFM) and Piezoelectric Force Spectroscopy (PFS) checked the piezoelectric and ferroelectric properties of the film.

## 1. Introduction

Multiferroics are materials in which at least two of the three ferroic orders, ferroelectricity, ferromagnetism or antiferromagnetism, and ferroelasticity coexist. Among multiferroics, perovskite bismuth ferrite (BiFeO_3_) and its derived systems are of special interest for keeping their properties in extreme temperature environments because of their Curie (*T_C_* = 1103 K) and Neel temperatures (*T_N_* = 643 K) well above room temperature [1,2]. In addition to ferroelectric and antiferromagnetic properties, BiFeO_3_ nanostructures, combined with graphene, show appealing photocatalytic activity [3,4]. Photoelectric, pyroelectric, and piezoelectric properties are the most studied and appealing characteristics, which make BiFeO_3_ an important material for energy harvesting applications [5]. The possibility of combining the above-mentioned properties in a single device, a hybrid energy harvester, makes BiFeO_3_ one of the most promising materials for the next generation of lead-free harvesters. For piezoelectric harvester, Pb(Zr_x_Ti_1-x_)O_3_ (PZT) has been commonly used [6,7], but the rising of environmental issues and questions on process sustainability has brought the light on lead-free perovskites, such as BiFeO_3_, LiNbO_3_ [8,9], and (K,Na)NbO_3_ [10], because of their encouraging capabilities for hybrid energy harvesting [11,12,13]. 

Different deposition techniques have been used to obtain thin films of BiFeO_3_, and their derived systems [14,15,16]. Most of the synthetic routes continue to rely on expensive single crystal substrates, such as SrTiO_3_, SrTiO_3_:Nb, and LaAlO_3_ [17,18], but recently flexible or even bendable substrates are investigated [19]. 

The main deposition techniques that have been applied to the production of BiFeO_3_ films are: chemical solution deposition [20,21], pulsed laser deposition (PLD) [22,23], sputtering [24], sol-gel [15,16], and metal organic chemical vapor deposition (MOCVD) [25,26,27,28]. So far, only sol-gel and sputtering have been applied to deposit BiFeO_3_ film used in a piezoelectric harvester [28,29], due to their simplicity and low operating temperature, which enable the use of a wide variety of substrates. The latest results for photovoltaic oriented devices have been obtained by PLD deposited films [23]. However, these techniques may have some drawbacks due to the substrate dimensions and difficulty in process scalability. MOCVD is a very appealing technique in terms of homogeneous and conformal deposition on large area substrates and easy possibility of scaling up, thus appointing itself as one of the best industrially applicable process. 

This paper aims to optimize a method for the fabrication of highly homogeneous BiFeO_3_ (from now on BFO) thin films on Si (001) that were buffered with an IrO_2_ layer, compatible with conventional industrial processes in order to create a functional hybrid energy harvester. Moreover, special attention has been given to the impact of the different deposition parameters on the quality of the films that were grown on Si. Si-based substrates present many advantages, the first one is the important cost reduction when compared to the widely spread single crystals. Subsequently, when considering that micro electro-mechanical systems (MEMs) microfabrication on Si implies well known protocols and techniques, keeping Si as base material for future functional structures is a key factor in lowering the cost and avoid the time-consuming process development for single crystals. 

X-ray diffraction (XRD) has been used for structural characterization, and field-emission scanning electron microscopy (FE-SEM) and energy dispersive X-ray analysis (EDX) have been used for the morphological and chemical analysis, respectively. Finally, local piezoresponse force spectroscopy (PFS) [30] and the domain mapping with piezoresponse force microscopy (PFM) have confirmed the piezoelectric/ferroelectric properties of the samples.

## 2. Materials and Methods 

### 2.1. Thin Film Deposition

Bi(phenyl)_3_ and Fe(tmhd)_3_ (phenyl = –C_6_H_5_, H-tmhd = 2,2,6,6-tetramethyl-3,5-heptandione), were mixed and used as a multicomponent precursor mixture. Bi(phenyl)_3_ and Fe(tmhd)_3_ were purchased from Strem Chemicals Inc. (Bischheim, France) and used without any further purification. Thin film depositions were performed in a customized, horizontal, hot wall MOCVD reactor with a 20° sample holder inclination. The bi-metallic mixture was placed in an alumina boat and heated at 120 °C. Argon and oxygen were used, respectively, as carrier and reactant gasses, by varying their flow from 150 sccm to 900 sccm (standard cubic centimeter per minute) for both species. The depositions were carried out in the temperature range 600–800 °C for 60 min. BFO films were deposited on a 10 mm × 10 mm Si (001) substrate coated with a 200 nm film of IrO_2_ acting, at the same time, as bottom electrode for piezoelectric characterization and as buffer layer between BFO and Si. 

### 2.2. Thin Film Characterisation

Analyses of crystalline phases were done through X-ray diffraction (XRD) measurements. θ–2θ XRD patterns were recorded in grazing incidence mode (0.8°) while using a Smartlab diffractometer (Rigaku, Tokyo, Japan), which was equipped with a rotating anode of Cu Kα radiation operating at 45 Kv and 200 Ma. The morphologies were examined through field emission gun scanning electron microscopy (FE-SEM), using a SUPRA VP 55 microscope (ZEISS, Jena, Germany). The films were analyzed by energy dispersive X-ray (EDX) analysis using an INCA-Oxford windowless detector (Oxford Instruments, Abingdon, UK) with an electron beam energy of 15 keV. 

Film roughness and ferroelectric properties were measured on an atomic force microscopy NT-MDT solver PRO with a conductive gold coated silicon cantilever, CSG10/Au purchased from NT-MDT (NT-MDT, Moscow, Russia). The BFO samples were grounded to the IrO_2_ bottom electrode and a good contact was obtained with silver paint.

## 3. Results and Discussion

### 3.1. Metal Organic Chemical Vapor Deposition (MOCVD) Grown BiFeO_3_ Films on IrO_2_/Si Substrate

**Figure 1:**
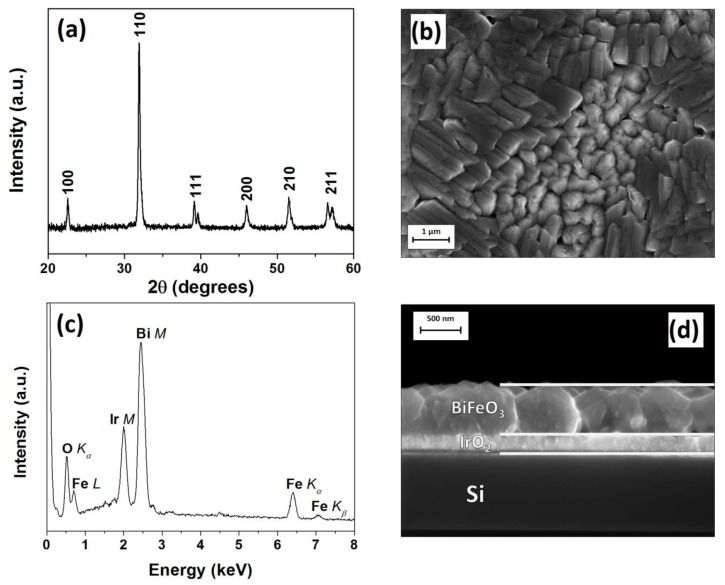
(**a**) X-ray diffraction (XRD) pattern, (**b**) field-emission scanning electron microscopy (FE-SEM) top view image, (**c**) energy dispersive X-ray analysis (EDX) analysis, and (**d**) FE-SEM cross section image of the BiFeO_3_ thin film deposited on IrO_2_/Si substrate.

Starting deposition experiments, as presently reported, are based on earlier works performed on single crystals SrTiO_3_ [26]. Good results were obtained in the temperature range 750–800 °C, with the best film quality and piezoelectric performance being obtained for the films that were deposited at 800 °C on SrTiO_3_. Thus, preliminary depositions have been carried out at 750 °C and 800 °C with Ar and O_2_ gas flows of 150 sccm for both species. Nevertheless, films that are deposited at 800 °C are not homogeneous and show delamination due to the effect of the high temperature on the IrO_2_ bottom layer, which causes its corrugation. Films deposited at 750 °C on a 10 × 10 mm^2^ IrO_2_/Si substrate, while using a susceptor with an inclination of 20°, show good properties in term of homogeneity and adhesion. The susceptor inclination has no effect on adherence, but it might have some effect on the homogeneity of the film. The sample structure has been checked using XRD and patterns have been recorded in the range 20–60° (Figure 1a) and are in agreement with the ICDD data (Card No 20-0169) based on the rhombohedal structure, space group R3c, of the BFO phase. 

The main diffraction peaks are observed at 2θ = 22.60°, 31.90°, and 39.15°, which are associated, respectively, with the 100, 110, and 111 reflections of the BFO phase, while considering a pseudocubic structure. BiFeO_3_ might be considered pseudocubic, having the rhombohedral structure cell parameters of a_rh_ = 3.965 Å and α_rh_ = 89.4° [2]. Thus, the deposited thin films are polycrystalline and comparison of the peak intensities with the database values indicates a slight preferential orientation along the <110> direction. 

FE-SEM has been used to monitor film morphology and, when coupled with energy dispersive X-ray analysis (EDX), to check chemical homogeneity and assess the elemental quantification in the films. The BFO films show a very uniform and dense morphology with massive and well coalesced grains. They adopt elongated shapes with two different preferential orientations (Figure 1b). EDX analysis shows chemical composition homogeneity on large sample areas. The sample average composition indicates a Bi:Fe ratio equal to 1 (Figure 1c). Film thickness has been also checked by several cross sections and shows an average value of 600 nm (Figure 1d). The IrO_2_ layer of 200 nm is clearly identifiable between the MOCVD grown BFO film and Si substrate (Figure 1d).

### 3.2. Impact of Gas Flows on BiFeO_3_ Film Growth

**Figure 2:**
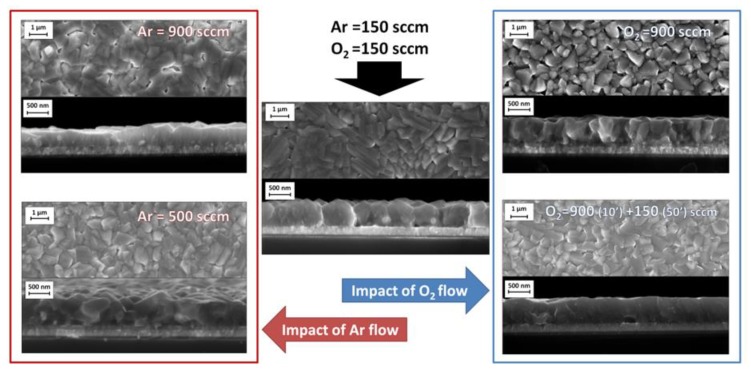
Comparison of BiFeO_3_ (BFO) thin films deposited under different conditions. BFO reference sample is in the center, on the left the impact of the argon flow and on the right the impact of the oxygen flow on BFO are presented. For every condition, FE-SEM top view and cross sections are reported.

Basic deposition parameters produced good quality films in terms of structure and composition. Nevertheless, the impact of the gas flows (either carrier or reactant) on the film morphology and quality is one of the major steps to develop a fully optimized process. Starting from the initial parameters, deposition temperature = 750 °C, Flow(Ar) = 150 sccm, Flow(O_2_) = 150 sccm, and duration 1h, the effect of the gas flow variation has been investigated by changing one gas flow (Ar or O_2_) and maintaining constant all of the other parameters to understand the impact of each one of them on the final product. In Figure 2, the results of BFO films deposited with diverse Ar or O_2_ flows are summarized based on the FE-SEM comparative images. 

The morphologies of films deposited with 500 sccm or 900 sccm Ar flows, keeping all the other parameters constant, are reported on the left side of Figure 2. The FE-SEM plan-view images show homogeneous samples with grains of 700–800 nm. The cross-section images show that the deposition at 500 sccm does not present major changes in terms of density and thickness with respect to the 150 sccm Ar deposited film. On the other hand, at 900 sccm, the cross section of the film indicates an important increase of film density, but the main drawback is that the film growth rate is significantly decreased when compared to our reference experimental conditions. The average growth rates drop from 9 nm/min to 4 nm/min. This variation might be related to a dilution effect, since the carrier flow increase determines the precursor dilution. The coupled influence of the carrier flow increase and precursor dilution is responsible for the lower growth rate and, consequently, for the formation of thinner but denser films at the highest carrier flow (900 sccm).

When considering previous results with argon, a significant flow of oxygen, 900 sccm, has firstly been tested. BFO growth rate is not limited by the high O_2_ flow and, at the same time, film top-view and cross section show a denser film composed of grains of about 800–850 nm (Figure 2, right side). According to these first observations, and to the promising impact that the high oxygen flow has on the film quality, a deposition experiment has been tailored while using a first step of 10 min. with Flow (O_2_) = 900 sccm followed by a second one of 50 min. with Flow (O_2_) = 150 sccm. The first step enables the creation of a seed layer with smaller nucleation sites, the second allows for these nuclei to grow, giving rise to coalesced grains forming a dense and homogeneous film. FE-SEM analysis (Figure 2, right side) confirms this observation, the grain size is smaller, and cross section shows an important diminution of the roughness and extremely dense BFO film. As expected, the growth rate is unchanged when compared to the reference sample. Thus, a visible improvement of the film quality is observed by simply varying the oxygen flow during the deposition of the film and, at the same time, the process remains straightforward without adding extra steps.

### 3.3. Impact of the Temperature on BiFeO_3_ Film

**Figure 3:**
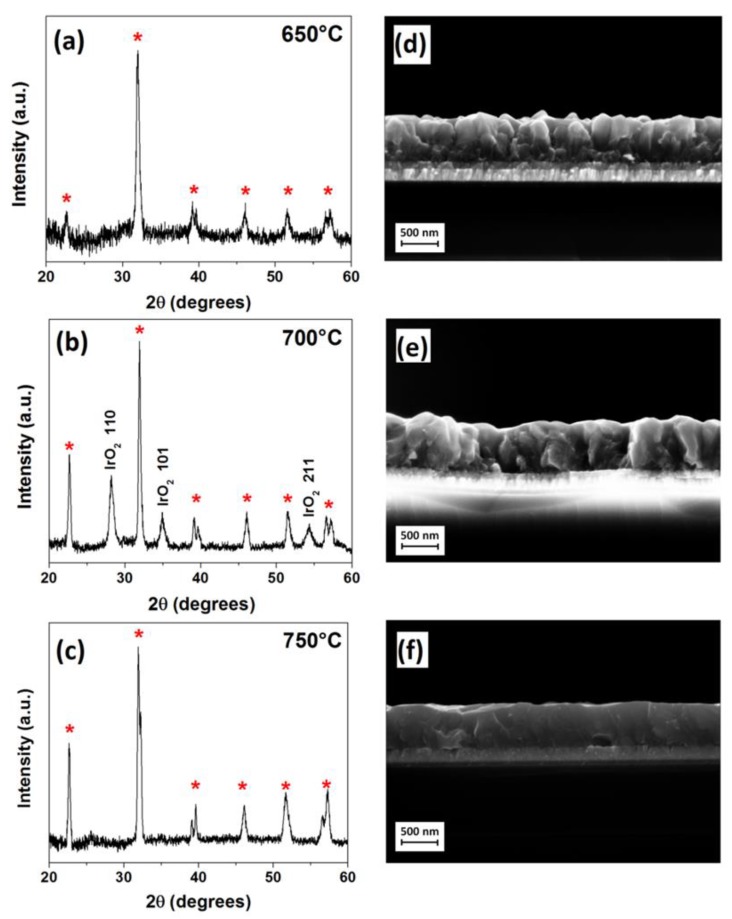
XRD diffraction patterns and FE-SEM cross section images of BFO thin films deposited at (**a**,**d**) 650 °C, (**b**,**e**) 700 °C, and (**c**,**f**) 750 °C.

Attempts to tune the sample orientation by changing deposition temperature have been done in the 600–800 °C temperature range with an Ar flow of 150 sccm and an O_2_ flow of 900 sccm for 10 min. and 150 sccm for 50 min. Depositions that were performed at 600 °C lead to a deterioration of film quality and homogeneity. FE-SEM image shows flat iron rich islands on the BFO film surface. Moreover, material quality and density are also heavily impacted by the low temperature process, coalescence is incomplete, and cracks are visible. At 800 °C, the IrO_2_ layer is no longer stable and it starts to interact with the film. The buffer layer corrugates in several points, forming bumps on the surface and leading to the delamination of the Si/IrO_2_/BFO structure. Defects that are caused by extreme conditions make the samples realized outside of the temperature range 650–750 °C, difficult to be analyzed, and compared to previous results. Within this ideal 100 °C deposition range, XRD patterns, as reported in Figure 3a–c, indicate that polycrystalline single phase BFO films are deposited. 

When comparing the various sample patterns, it has been observed that a preferential orientation competition takes place between the (100) and (110) planes, but no complete orientation is observed, whatever the deposition parameters. The cross-section images (Figure 3d–f) of the films deposited in this temperature range show similar thicknesses, thus pointing to similar growth rates. Specifically, growth rates have been evaluated every 50 °C between 600 °C and 750 °C, yielding growth rates of 7, 10, 11, and 10 nm/min., respectively, for 600, 650, 700, and 750 °C.

**Figure 4:**
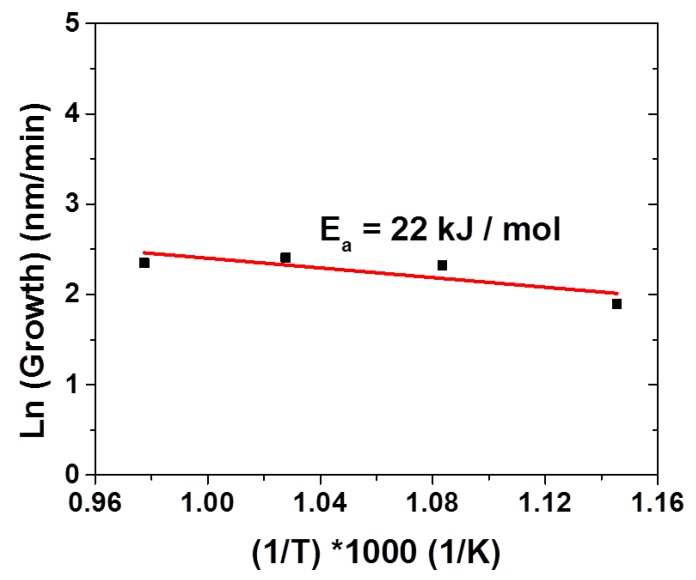
Arrhenius plot of the ln growth rate vs. 1000/T for the BiFeO_3_ MOCVD process starting from the Bi(phenyl)_3_ and Fe(tmhd)_3_ precursors.

This independence between film thickness and substrate temperature in the 600–750 °C range seems to point to a mass transport regime. Figure 4 represents the plot of the ln growth rate vs. 1000/T. The apparent activation energy of 22 kJ/mol, as derived from the Arrhenius plot, clearly indicates that, in the used deposition temperature range, BFO film growth occurs in the mass transport-limited regime.

### 3.4. Functional Properties

**Figure 5:**
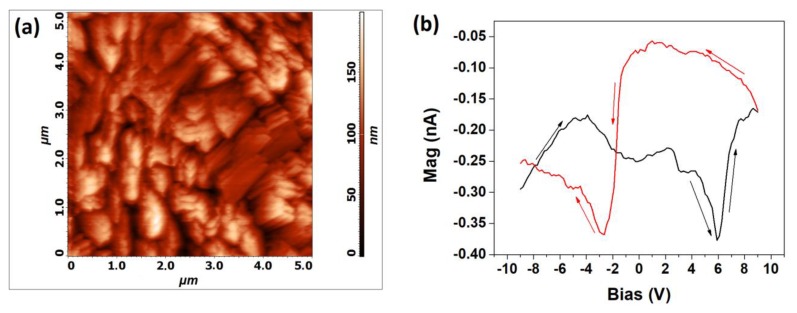
(**a**) Atomic force microscopy (AFM) scan of a 5 µm × 5 µm area and (**b**) butterfly loop (the “black” curve corresponds to the −9 V to +9 V scan and “red” curve corresponds to the +9 V to −9 V) of the “as deposited” BFO thin film on Si obtained by PFS.

BFO thin film topography on the IrO_2_ buffer layer has been recorded by a classical contact mode vertical atomic force microscopy (AFM) scan (Figure 5a). The 5 µm × 5 µm investigated area exhibits the same morphology as the one that was observed by FE-SEM (Figure 1) with a root mean square (RMS) roughness of 28.6 nm. The piezoelectric property of the deposited film on the IrO_2_/Si substrate has been investigated through piezoresponse force spectroscopy (PFS). Piezoresponse (in terms of the amplitude of the out of plane displacement, Mag), as a function of the alternating voltage, showed the typical butterfly loop [30,31] for an applied bias from −9 V to 9 V between microscope cantilever tip and BFO bottom electrode IrO_2_ (Figure 5b), indicating the piezoelectric behavior of the film.

In Figure 5b the unit for the Mag is nA, because the instrument measures the vertical displacement from the photocurrent of the laser beam reflected by the displaced tip. In particular, the tip displacement is proportional to the difference between the photocurrent incident on different photodiode sections. Figure 5b shows a hysteresis loop, since, after applying a −9 V bias and then reducing the bias until 0 V (black curve), the piezoresponce is different from that observed when applying a +9 V bias and then reducing it to 0 V (red curve).

**Figure 6:**
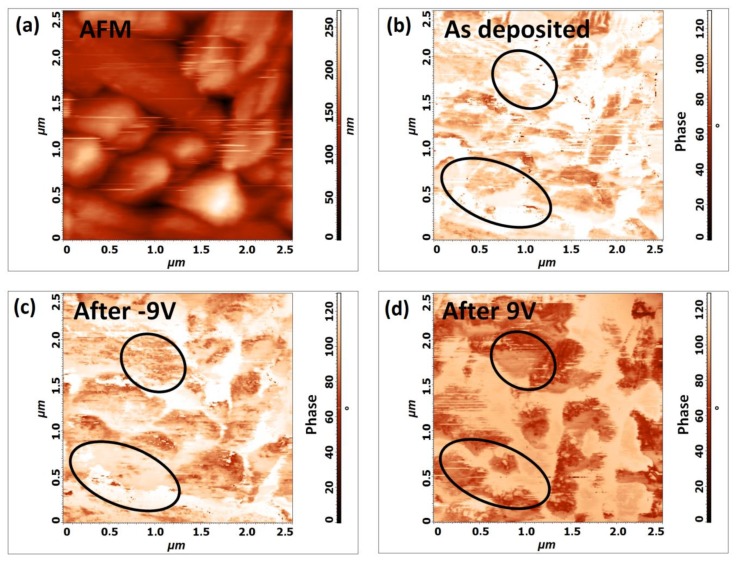
Ferroelectric domains switching: (**a**) AFM image of the scanned 2.5 µm × 2.5 µm area and (**b**) phase scan of the “as deposited” BFO film; (**c**) phase scan of the BFO film after the application of a −9 V bias; and, (**d**) phase scan of the BFO film after the application of a +9 V bias.

Following this first observation, a study of ferroelectric domain switching [32] has been carried out on a 2.5 µm × 2.5 µm area. Figure 6a reports the AFM topography of the scanned area. At first, a PFM image of the ferroelectric domain of the “as-deposited” BFO film was obtained in terms of phase difference between the vertical piezoresponse signal and an applied alternating voltage, before the application of any bias voltage (Figure 6b). Subsequently, to observe the switching of the domains, a bias voltage of −9 V was applied to the entire area through the scanning tip and map of the ferroelectric domains was recorded with a 0 V bias voltage (Figure 6c). Subsequently, a similar PFM image was obtained at 0V (Figure 6d), after the application of a +9 V bias. The polycrystalline nature of the film might limit phase scan interpretation and, indeed, the measured signal is the average of all the ferroelectric domains that were placed between the cantilever tip and the bottom electrode. Very few differences between domain phases of the “as prepared” BFO film and the film after application of the −9 V bias are visible (Figure 6c). On the other hand, the impact of the application of +9 V bias on the film polarization was much more important (Figure 6d), indicating the switching of several ferroelectric domains. As an example, the material has a visible response after bias voltage application because of domains switching when comparing the circled zones on Figure 6b–d. 

PFS and PFM confirmed the piezoelectric properties of the as deposited BFO film. Furthermore, even if sample polycrystallinity might limit ferroelectric mapping, domains switching can be observed by reducing the working area, thus confirming the functional properties of the BFO thin film on Si.

## 4. Conclusions

In conclusion, BFO films have been successfully deposited by MOCVD on silicon substrate, with an IrO_2_ bottom electrode, acting as a buffer layer since it can stand high temperatures. The morphology, density, thickness, and Bi:Fe ratio in the films are homogenous on the whole sample surface of 10 mm × 10 mm. The BFO thin films show different growth orientations, but no specific relationship has been found between orientation and experimental conditions. Various experiments indicate that the optimal deposition temperature range is between 650 °C and 750 °C with a fixed argon flow of 150 sccm and the use of high oxygen flow, 900 sccm for 10 min., in order to induce the formation of numerous BFO nucleation sites, and 150 sccm for 50 min. to trigger the growth of a denser film with smaller grains when compared to the other investigated conditions. 

Thus, the BFO films can be successfully deposited on Si at a lower temperature and in a more cost-effective process, with respect to the previously reported methodologies. Moreover, the present approach offers the major advantage to be easily scalable and the use of IrO_2_, as a conductive oxide, gives the opportunity for future characterizations and device microfabrications. Finally, it can be pointed out that the material quality and production cost of lead-free perovskites, as BFO, are key points for the scaling-up development of a new generation of hybrid energy harvesting devices. The MOCVD approach, as presently reported, answers to both demands and it is compatible with the current technologies.
